# How to implement pairing assistance during fighting COVID-19 in China: collaborative governance between local governments under the authoritative regulation

**DOI:** 10.3389/fpubh.2024.1417832

**Published:** 2025-01-09

**Authors:** Changwei Wei, Jiaxi Xu, Zhixiang Wang, Huangyue Wu, Juan Wang

**Affiliations:** ^1^School of Public Policy and Management, China University of Mining and Technology, Xuzhou, China; ^2^School of Political Science and Public Administration, Wuhan University, Wuhan, China; ^3^School of Public Administration, Jilin University, Changchun, China

**Keywords:** authoritative regulation, collaborative governance, pairing assistance, COVID-19, public crisis

## Abstract

**Background:**

The pairing assistance policy represents a distinctive instrument utilized by the Chinese government to address major public crises. This study examines the development of a pairing assistance policy by the Chinese Government through its central authority to foster collaborative governance among local governments in areas affected by COVID-19.

**Methods:**

The aim of the study was to gain a clear understanding of how the policy of pairing assistance in public health emergencies is successfully implemented through the top-down application of authority. A case study design was used as a methodology to present an explanatory framework for implementing pairing assistance policies during major public crises. We focus on the operational process of pairing assistance, using the assistance provided by Jiangsu Province to Huangshi City in Hubei Province as an illustration.

**Results:**

This paper finds that responding to a crisis requires the guidance of a central authority and the cooperation of local governments. The process is driven by three key factors: the vertical intervention of the crisis, the inevitability of horizontal cooperation and the policy allocation and incentives of the bureaucracy. The three stages of co-operative governance based on authoritative regulation work together in a step-by-step manner to enhance the effectiveness of crisis response.

**Conclusion:**

The results of the study indicate that collaborative governance under the authoritative regulation is the main reason why provincial counterpart support mechanism plays a great role in COVID-19. This study is the first to approach the study of pairing assistance from the perspective of government authority. It broadens the research horizon of local government cooperation and provides a model for future collaboration.

## 1 Introduction

“Pairing assistance” is a distinctive regional cooperation policy unique to China, designed to provide targeted assistance to underdeveloped regions or those facing major crises that they cannot overcome independently. Pairing assistance is a governance mechanism with Chinese characteristics for the horizontal resource transfer and cross-border cooperation in China (1). The mechanism of pairing assistance is a living practice of cross-sector, cross-level and cross-regional coordination in emergency management (2). Its effectiveness was particularly demonstrated during the unprecedented challenge of COVID-19. At the dawn of 2020, the outbreak of COVID-19 in Wuhan, China. In this context, the Chinese government launched an initiative called “Pairing Assistance for Hubei.” This initiative orchestrated a strategic allocation of resources by enlisting the support of various provinces to assist 16 cities in Hubei Province (3).

A notable example of this support policy was the dispatch of 310 medical personnel from 10 cities in Jiangsu Province to Huangshi City, Hubei Province, on 11 February 2020 (4). After days of work, the Jiangsu provincial medical team successfully treated 880 cases, achieving a cure rate of over 96% (5). The impact of this intervention was profound, with Huangshi City reporting zero confirmed cases, suspected cases, and close contacts of local COVID-19 cases on 27 March (6). The policy of pairing assistance has proven to be a critical component in China’s arsenal against the COVID-19. Faced with a rapid surge in cases and an initially limited effective treatment or isolation strategies, the rapid activation of the pairing assistance policy by the Chinese government was an example of an adaptive and resilient response to the crisis.

Pairing assistance is a policy instrument that is unique within the Chinese governance framework. While scholars have studied local intergovernmental cooperation and regional mutual aid during crises, the Chinese model of pairing assistance stands in contrast to the experience of other nations, such as the United States, a country of comparable economic scale. Studies have indicated that cooperation between local governments in the U.S. has been less effective during crises (7). For example, Mallinson’s analysis of the dynamics between federal and state governments during the COVID-19 outbreak revealed a landscape characterized by independent action and a lack of cohesive strategy, leading to the uncontrolled spread of the virus (8). Joyce and Suryo’s examination of the legal and financial responses to COVID-19 in the United States highlighted the inadequacy of federal policies and resources to contain the pandemic (9). They found that COVID-19 had continued to spread despite a series of measures enacted by the US federal government and the corresponding financial appropriations passed by Congress. South Korea is one of the few countries that has effectively controlled the COVID-19 in early stages. As a neighboring country to China, South Korea shares a similar crisis culture with China (10). The Korean government has responded to COVID-19 with greater rigor than many other countries. Scholars such as Kim et al. have examined South Korea’s crisis response system and found a comprehensive national framework developed through inter-agency cooperation and expert consultation (11). Yoo et al. have further highlighted the roles and responsibilities of various government and private sector agencies in South Korea’s response, emphasizing the importance of strong leadership and coordination (12). They emphasize the importance of strong national leadership and coordination in this process. Chen et al. explained the process and effects of China’s “pairing assistance” policy during COVID-19, arguing that “the implementing of pairing assistance is a turning point in China’s fight against epidemics” (13). Pan et al. examined the activation of the pairing assistance mechanism by the Chinese government in the face of the critical emergency posed by COVID-19, deploying a contingent of over 10,000 medical personnel nationwide to support Hubei Province (14).

Through a case study analysis of Jiangsu Province’s assistance to Huangshi City, this paper identifies collaborative governance under authoritative regulation as a critical facilitator of the pairing assistance policy. The findings highlight the potential of such a policy for effective crisis management, particularly in contexts where resources are scarce and the urgency of the situation demands a coordinated and decisive response.

## 2 Materials and methods

Numbers of studies have attempted to identify the logic and potential of collaborative disaster response through case studies. Smith and Dowell explored the challenges of coordination by examining a railway accident in the UK and identified two key sources of complexity (15). Similarly, Raju and Van Niekerk explored issues of public sector coordination and sustainable disaster recovery through a case study of the Eden District Municipality in South Africa (16). Steigenberger’s work provided insights into disaster response coordination through an analysis of multi-agency collaboration in different disaster scenarios (17). Kapucu et al. investigated the collaborative network dynamics between non-established relief groups (NERGs) and other emergency management agencies in the context of Hurricane Irma (18). As Chao has articulated, the case study design is favored for its capacity to provide researchers with nuanced understanding of particular individuals, problems, or unique situations through an intensive and in-depth examination of the phenomenon (19). The utility of case studies in disaster research is well-acknowledged, as they facilitate a detailed analysis of the complex interplay between multiple agencies and levels of governance in disaster response. In keeping with this scholarly tradition, this paper constructs a comprehensive database, the research gathered publicly available information from government websites, relevant news reports, and case data accessible through local health commissions. Through an in-depth examination of textual and quantitative information, this study re-evaluates the introduction, operation, and rationale of the pairing assistance policy, thereby contributing to the understanding of multi-agency and multi-level interactions in disaster response.

The study selected the case of Jiangsu Province’s assistance to Huangshi City, Hubei Province, firstly because the COVID-19 was a representative outbreak which, as a spillover crisis, had far-reaching consequences for the whole country and the world (20). China’s efforts to control the COVID-19 were a comprehensive process that highlighted the roles and relationships between the central and local governments (21, 22). This process provides valuable insights into Chinese politics and government. At the same time, COVID-19 attracted a high level of social concern (23), and the high level of information disclosure had a direct and far-reaching impact on economic and social development (24). The Chinese government was able to control the spread and number of new infections within a short period of time, at a time when the Spring Festival in China coincided with a sharp increase in population movement, demonstrating the effectiveness of the pairing assistance policy. The central government designated Jiangsu Province as one of the regions to support Huangshi City, following the principle that the regions should be matched based on their capacities to provide assistance and the extent of their needs. In previous pairing assistance initiatives, Jiangsu Province, as a developed province in China’s eastern coastal region, was often paired with regions more severely affected by disasters. On 9 February, 2020, Huangshi City reported 805 confirmed cases of COVID-19. The city’s proximity to Wuhan, coupled with its convenient transportation links, meant that a significant number of Huangshi residents worked and lived in Wuhan. Furthermore, the relatively lower level of economic development in Huangshi resulted in a weaker healthcare infrastructure. The return of a large number of migrant workers to Huangshi City further exacerbated the increase in local infections (25). These factors collectively informed our decision to select this case as the subject of our study.

We divide the operation of the pairing assistance policy into three stages, describing the ways in which the Chinese government orchestrates the emergency response behavior of local governments through central intervention and control, culminating in a model of collaborative governance. This paper aims to synthesize relevant theories and research to analyzes the outcomes of collaborative governance under authoritative regulation, building on the successful results of pairing assistance.

## 3 Results

### 3.1 Stage 1: launch

In December 2019, an outbreak of the COVID-19 epidemic occurred in Wuhan, Hubei Province, and rapidly spread throughout China, which brings a huge impact on China (26). The outbreak of this highly infectious disease g attracted a great deal of national and international attention. However, this concentration of resources also inadvertently strained the control and prevention capacities of other cities within Hubei Province, highlighting the complex challenges of resource allocation during large-scale public health emergencies. As shown in Table 1, due to geographical proximity and the government restrictions on inter-provincial movement (27), there was a large influx of people from Wuhan to other urban areas in Hubei Province (28). This migration pattern has resulted in the reporting of confirmed COVID-19 cases in other cities in the province, with an observed acceleration in the rate of case increase (29). Despite this, the attention and assistance received by the rest of Hubei Province has been markedly limited in comparison to Wuhan. As the COVID-19 outbreaks in these cities, it became clear that local communities were insufficiently equipped to mount an effective response on their own. The combination of internal pressures and uneven external support acted as a catalyst for local governments to seek additional assistance.

**Table 1:** The proportion of people departing from Wuhan to other cities in Hubei Province from January 10th to January 22nd (%).

Xiaogan	10.94	13.80	13.47	12.04	12.64	13.76	12.57	12.56	13.16	14.47	14.24	13.87	13.34
Huanggang	10.52	11.75	11.19	11.39	12.56	13.30	13.35	14.21	14.87	12.20	12.45	13.50	12.95
Jingzhou	5.74	5.91	5.74	5.80	5.84	6.03	6.00	5.93	6.29	6.93	7.29	7.17	7.80
Xianning	5.22	5.95	5.32	4.94	4.97	5.10	4.96	5.07	5.14	4.95	4.75	4.77	4.38
Ezhou	4.12	4.53	4.83	4.77	4.36	4.10	4.04	4.23	4.39	3.91	3.53	3.28	3.26
Xiangyang	4.12	3.92	3.66	3.72	3.68	3.44	3.44	3.58	3.63	3.81	4.08	4.44	4.74
Huangshi	3.42	3.81	3.74	3.70	3.69	3.68	3.84	3.94	4.21	3.75	3.70	3.74	3.40
Jingmen	2.85	2.95	2.72	2.76	2.73	2.82	2.81	2.75	2.96	3.31	3.59	3.76	3.91
Suizhou	2.52	2.71	2.65	2.67	2.68	2.82	2.89	2.98	3.11	3.21	3.38	3.54	3.66
Xiantao	2.38	2.81	2.80	2.66	2.59	2.88	2.80	2.76	2.91	3.07	3.11	3.23	3.19
Yichang	3.08	3.24	2.76	2.43	2.35	2.48	2.50	2.54	2.69	2.95	3.05	3.05	3.49
Tianmen	1.47	1.76	2.01	1.77	1.95	1.97	2.07	1.95	2.10	2.33	2.43	2.28	2.28
Enshi	2.12	1.92	2.11	1.83	1.89	1.79	1.80	1.74	1.80	1.74	1.87	1.83	1.80
Shiyan	2.02	1.85	1.88	1.76	1.65	1.60	1.50	1.56	1.65	1.84	1.97	2.00	1.99
Qianjiang	1.10	1.14	1.28	1.18	1.04	1.01	1.03	1.02	1.03	1.04	1.17	1.19	1.43
	1.10	1.11	1.12	1.13	1.14	1.15	1.16	1.17	1.18	1.19	1.20	1.21	1.22

The implementation of the pairing assistance policy was gradually developed under conditions of restricted population mobility. As large numbers of infected people appeared in other cities in Hubei Province, the central government, in an effort to contain the spread of the disease, enacted measures to restrict population mobility, including the imposition of city lockdowns. Pairing assistance served as a complementary policy to these restrictions. It facilitated the transport of medical resources from other provinces to Hubei Province without compromising the effectiveness of disease control and prevention efforts, thus providing a solution to the mobility restriction measures.

### 3.2 Stage 2: decision

During the decision stage, the central government of each country must play a key role (30). The Chinese central government has multiple roles in the entire process of pairing assistance, from decision-making to implementation. By regulating with authority, it has continuously promoted the implementation of the pairing assistance policy. First, as a decision-maker, the Chinese central government takes responsibility for responding for responding to policy appeals from local governments. Upon receiving requests for assistance, the central government conducts a comprehensive assessment of the actual circumstances to determine the appropriateness of a response. Second, as a coordinator, the central government is tasked with making decisions based on the specific conditions in each region. This involves deploying corresponding policies, coordinating relevant relationships, and orchestrating the allocation of resources, thereby acting as the coordinator of policy implementation. Third, the central government also plays a critical role as a supervisor in the implementation of the pairing assistance policy. It is responsible for monitoring the implementation efforts of each region and, if necessary, providing rewards or penalties to ensure the effective implementation of the policy. This supervisory function is essential in guaranteeing that the pairing assistance policy is executed with efficacy.

The COVID-19 outbreak coincided with Chunyun, the period of mass migration for the annual Chinese Spring Festival (31). During this time, a large number of people left Wuhan for other parts of Hubei and other provinces in the country, which may have contributed to the spread of the virus. The Chinese government is facing a challenging internal environment due to a combination of factors: China’s economy has come to a standstill due to the strict containment and prevention measures implemented (32). The outbreak has spread throughout the country, increasing fears of a pandemic and resulting in considerable public and media attention. On 20 January, Chinese President Xi Jinping made important instructions on the COVID-19, emphasizing the importance of prioritizing people’s safety and health and resolutely controlling the epidemic (33). The safety of citizens’ lives is paramount in the prevention and control of COVID-19.

In 2018, the gross domestic product (GDP) of Wuhan was the highest in Hubei Province, equal to the combined GDP of the second to sixth largest cities in the province. This economic disparity is reflected in the healthcare sector, where significant differences in resource allocation are evident. Wuhan, with a permanent population of 11.081 million, has more than thirty A-level tertiary hospitals, while Xiangyang, the second largest city in the province with a population of 5.5 million, has only five such hospitals. This was evidenced by the high number of cases and deaths in cities such as Huanggang, Xiaogan, Ezhou, Tianmen, and Huangshi. These regions, which have a high proportion of migrant workers in Wuhan, were severely affected by the outbreak. Huanggang and Xiaogan consistently occupied the second and third positions in terms of confirmed cases for an extended period. Tianmen, which is at the lower end of the economic spectrum in Hubei, had a significantly higher mortality rate than Wuhan due to its relatively underdeveloped healthcare facilities. In response to Hubei’s request for assistance, the central government took 3 days to establish a provincial counterpart support mechanism (Table 2), with 19 provinces supporting 16 cities in Hubei Province, in the form of one province being responsible for one city (34). The elevation of pairing assistance to a “political task “highlights the central government’s emphasis on responding to COVID-19.

**Table 2:** Table of cities in Hubei with pairing assistance from each province released by the National Healthcare Commission (Data up to February 10, 2020).

Cities	Confirmed Cases	Supporting provinces
Xiaogan	2,541	Chongqing, Heilongjiang
Huanggang	2,252	Shandong, Hunan
Suizhou	1,049	Jiangxi
Jingzhou	1,045	Guangdong, Hainan
Xiangyang	1,019	Liaoning, Ningxia
Huangshi	805	Jiangsu
Yichang	749	Fujian
Ezhou	725	Guizhou
Jingmen	641	Inner Mongolia, Zhejiang
Xianning	507	Yunnan
Shiyan	481	Guangxi
Enshi	187	Tianjin
Xiantao	416	Shanxi
Tianmen	217	
Qianjiang	85	
Shennongjia	10	Hebei

### 3.3 Stage 3: implementation

Jiangsu Province, as one of the designated entities tasked with executing the central government’s pairing assistance policy, has been instrumental in providing support to Huangshi City in Hubei Province. The commitment to this policy was underscored during a meeting on 10 February, when the Jiangsu Provincial Government emphasized, “We stand shoulder to shoulder, hand in hand with Huangshi, committed to overcoming COVID-19.” The Jiangsu provincial government has demonstrated its acceptance to this policy through both political rhetoric and concrete actions. This is evidenced by the following initiatives: First, in response to the pairing assistance policy, Jiangsu Province conducted a special meeting to strategize and deploy the necessary measures for its implementation. Second, the Governor of Jiangsu Province personally saw off the medical team, underscoring the province’s dedication to the cause. Third, the provincial government established a high-level emergency command center, which was directly stationed in Huangshi City, to coordinate the assistance efforts effectively.

In the response to the COVID-19, the 13 cities of Jiangsu Province demonstrated a high level of inter-regional cooperation, with hundreds of medical personnel from 13 cities participating in the medical team that assisted Huangshi City (Table 3). From 11 February, when the first group of medical personnel from the Jiangsu-Huangshi medical team left, until 13 April, when all the medical teams returned, the Jiangsu-Huangshi medical team worked tirelessly for almost 2 months. They were stationed at eight designated hospitals, including the Huangshi Central Hospital, where they participated in the treatment of 419 critically ill patients, 860 severe cases, 5,884 moderate cases, and 1,209 mild cases. After the arrival of the Jiangsu-Huangshi medical team, the number of new cases in the local area decreased significantly. Of the 1,015 cases reported in Huangshi City, 86.1% were pre-existing cases before the team arrived (35). Local NGOs also play an important supporting role. For example, volunteer associations in Wuxi have established a partnership with volunteer associations in Huangshi, while Nantong and Huangshi have collaboratively organized the “Jiangsu-Huangshi Festival” in a virtual format. Through these frequent interactions, the local governments are not only actively demonstrate their commitment to central government but also delivering a sense of warmth to the local communities. This dynamic engagement underscores the proactive approach of local governments in implementing the pairing assistance policy, thereby highlighting the multifaceted dimensions of policy execution that encompass both practical support and symbolic gestures of solidarity.

**Table 3:** Composition of the first medical team dispatched by Jiangsu Province to assist Huangshi City.

Composition of medical teams	
Cities	Nanjing, Xuzhou, Changzhou, Nantong, Lianyungang, Huai’an, Yancheng, Yangzhou, Zhenjiang, Taizhou
Medical Institution	Jiangsu Provincial People’s Hospital, Jiangsu Provincial Hospital of Traditional Chinese Medicine, Jiangsu Provincial Centre for Disease Control and Prevention, The Second Affiliated Hospital of Nanjing Medical University, Jiangsu Provincial Organs Hospital, Jiangsu Provincial Cancer Hospital, Jiangsu Provincial Hospital of Integrative Medicine, Jiangsu Provincial Hospital of Traditional Chinese Medicine, Jiangsu Provincial Hospital of Traditional Chinese Medicine, The Second Hospital of Traditional Chinese Medicine of Jiangsu Province, The Affiliated Hospital of Xuzhou Medical University, The First Affiliated Hospital of Soochow University, The Affiliated Children’s Hospital of Soochow University, The Affiliated Hospital of Nantong University, The Affiliated Hospital of Southeast University The Affiliated Yat-Fu Hospital of Nanjing Medical University, Jiangsu University Hospital, Yangzhou University Hospital
Staff	Including 103 doctors, 200 nurses, 4 public health personnel and 3 cadres assigned by the Jiangsu Commission of health, totaling 310 personnel
Age	The oldest is 60 years old and the youngest is 23 years old

## 4 Analysis

The previous case provides an overview of common scenarios in which local governments have taken over directive control from higher authorities in the midst of crises, thereby effectively implementing local cooperative governance mechanisms. This section aims to refine the framework of collaborative governance between local governments under authoritative regulation. This refined framework is posited to elucidate the factors that contribute to the successful execution of pairing assistance policy, thereby providing a structured understanding of the underlying dynamics of policy implementation in contexts of crisis management.

### 4.1 Authoritative regulation: vertical intervention in crisis

Authoritative regulation is the process by which the central or higher-level governments use the bureaucratic pressure to promulgate policy on particular issues. This process facilitates cooperation with local governments by reallocating authority from the local to the central bureaucracies (36). In China, where the central government has significant influence over key decision-making processes, the implementation of cooperative governance at the local level is largely dependent on directives issued from higher levels of government. To facilitate the way in which local governments work together to address public issues or pursue specific policy objectives, the central government uses a number of measures.

The first policy tool is to focus the attention of governments at all levels. The involvement of local governments in inter-regional governance has the potential to effectively dismantle the traditional management and control governance model that is confined within the boundaries of administrative divisions (37). However, the formation of cooperative relationships between local governments is challenged by a dilemma of “transaction costs,” which complicates the establishment of the relationships. Crisis is not only a material fact that affects society (38), but also draws attention to the importance of collaborative governance. The occurrence of crisis enables the central government to focus the attention of governments at all levels, facilitating intervention and the optimization of collaborative governance for major crises across regions.

The second stage is the emphasis on political discourse. The establishment and maintenance of government authority is crucial to the formation of authoritative regulation, which is continuously interpreted, upheld, and solidified within the practice of national governance. While adapting to the changes of the times, the Chinese government has enhanced the central government’s influence over local jurisdictions and strengthened its control through the innovation of systems and mechanisms. In achieving this objective, it has also ensured the vitality of local governments. The tax-sharing reform of the 1990s serves as an example of this governance practice.

The third element concerns the establishment of principal-agent relationships. China’s decentralization model exhibits characteristics of a principal-agent relationship (39). The central government coordinates and plans national public affairs, and regulates policy implementation based on actual diverse choices to achieve strategic objectives. Local governments, functioning as extensions of the central government, primarily act as the entities responsible for policy execution. In the process of bureaucratic coordination for crisis prevention and control, the Chinese government’s approach to governance predominantly adopts a “pressure-based system combined with campaign-style governance,” which swiftly and effectively mobilizes various elements of governance (40).

### 4.2 Collaborative governance: horizontal synergies in a crisis

According to Chris et al., “Collaborative governance, as it has come to be known, brings public and private stakeholders together in collective forums with public agencies to engage in consensus-oriented decision making” (41). Kirk et al. argued that collaborative governance broadly as the processes and structures of public policy decision making and management that engage people constructively across the boundaries of public agencies, levels of government, and/or the public, private and civic spheres in order to carry out a public purpose that could not otherwise be accomplished (42). In the Chinese context, collaborative governance is a model that spans the boundaries between government and society, and aims to protect the public interest while achieving a win-win situation for all parties involved. Previous experiences have shown that relying solely on traditional national approaches is inadequate in responding to complex crises (43). The characteristics of an infectious public health emergency have a significant impact and can cause spillover effects (44). This type of public crises often requires collaborative governance, which needs institutional mechanisms for collaboration, multi-level and effective cross-sectoral leadership (45).

The basic premise for collaborative governance among local governments is, first, the shared vulnerability to trans-boundary crises. The governance of public crises inherently possesses a cross-regional character. The public crises often arise from the confluence of multiple risk factors, rendering them intricate, diverse, and unpredictable (46). Such crises can manifest both within specific regions and across regional boundaries, with the latter transcending the confines of traditional administrative jurisdictions and necessitating collaborative regional governance strategies. Second, the uneven distribution of governance resources presents significant challenges in public crises. Effective crisis management demands substantial governance resources, which are frequently in short supply across different regions. The disparity in the distribution of governance resources across regions is pronounced (47), with less developed regions exhibiting significantly lower capacity for risk bearing compared to their more developed counterparts, thereby rendering them more susceptible during crisis response efforts. Meanwhile, public choice theory posits the existence of an “economic man” (48), implying that local governments are likely to pursue cost minimization and may adopt a “free rider” strategy in the face of crises. Consequently, the governance of crises necessitates the coordination of intergovernmental relations to ensure a cohesive and effective response.

The second element to consider is the strong foundation for cooperation inherent in China’s political system. The systemic advantages of the Chinese model, characterized by the “national system,” the ethos of “the whole nation working in unison,” and the capacity to “marshal the resources necessary to undertake grand endeavors,” empower the Chinese government to mobilize administrative resources and public authority swiftly (49). Concurrently, the establishment of a series of laws and regulations, such as the Emergency Response Law of the People’s Republic of China and the National Emergency Response Plan for Public Health Emergencies, signifies the institutionalization of pairing assistance in public crises. On the other hand, China’s cultural tradition, epitomized by the adage “Sailing in the same boat and helping each other,” boasts a rich historical heritage, and the principle of “When one side is in distress, all sides offer support” has long been a societal consensus in China. Culture plays a key role in crisis response (50). This collectivist approach to disaster response provides a significant cultural and societal foundation for the application of pairing assistance, thereby facilitating its practical implementation.

The third foundational element is the practical experience. David Miller noted that “within a community, the likelihood of cooperation is enhanced by a higher level of trust” (51). The concept of paring assistance has been an important part of the Chinese Government’s policy agenda. Over the course of several decades, the practice of pairing assistance in ethnic and border areas has evolved, with notable examples including the “Great Western Development Strategy” (52). Similarly, pairing assistance for major projects was manifested in the implementation of the Danjiangkou Reservoir Project (1973) and the Three Gorges Dam Project (2003) (53). Pairing assistance in disasters and emergencies was also mobilized in response to the outbreak of Avian Influenza A (H1N1) outbreak in 2009. The policy program of pairing assistance, a unique feature of China’s governance approach, has been implemented over many years and has accumulated considerable practical experience.

### 4.3 Driving mechanisms: policy allocation and incentive in the bureaucracy

The driving mechanism underlying the governance framework can be divided into two distinct components. The first component is the policy allocation within the bureaucratic structure. Bureaucracy has been characterized as a complex, hierarchical system of governance designed for the purpose of policy decision-making (54). Bureaucratic institutions operate under the regulatory authority of the central government, which has the power to define issues, allocate attention to different issues, and control and realign key tasks. In times of crisis, central government facilitates the implementation of mating support policies by allocating resources and reinforcing policy directives through increased vertical intervention.

The second component is the incentives and constraints inherent in the system. n China, career advancement within the bureaucracy is influenced by the institutional landscape of political centralization (55). The central government directly influences the promotion of local government officials (56). Within the Chinese governance apparatus, higher-level governments evaluate the performance of critical policy tasks and key policy decisions when considering the appointment or dismissal of officials. Historically, during major public crises, officials are given increased responsibility and a high degree of trust (57). Exceptional performance in crisis management can serve as a direct pathway to promotion. The mechanism of incentives and constraints for lower-level governments is strategically designed by higher-level governments to “reward diligence and punish indolence” through appointments and dismissals. Given the measurable and highly visible outcomes of pairing assistance missions, their successful implementation can easily serve as a basis for career advancement.

### 4.4 Mechanism update: collaborative governance under the authoritative regulation

Bryson and colleagues note that multisectoral governance approaches have matured in response to major challenges such as natural disasters, rising inequality and deteriorating health systems (58). These approaches are often conceptualized under the rubric of collaborative governance (59). Pairing assistance becomes essential when dealing with crises that have impacted or have the potential to affect multiple regions or even the whole country. The provision of assistance in the wake of major public crises is not just a consequence of central government regulation; it is also a practical imperative for local governments to collaborate in addressing these crises. Central government can achieve effective governance of public crises through authoritative regulation that encourages cooperative behavior between local government. Defining specific governance objectives can mitigate the tendency of local governments to engage in speculative behavior within the framework of authoritative regulation. Authoritative regulation can serve to complement and facilitate horizontal cooperation between local governments, playing a pivotal role in its enablement.

**Figure 1:**
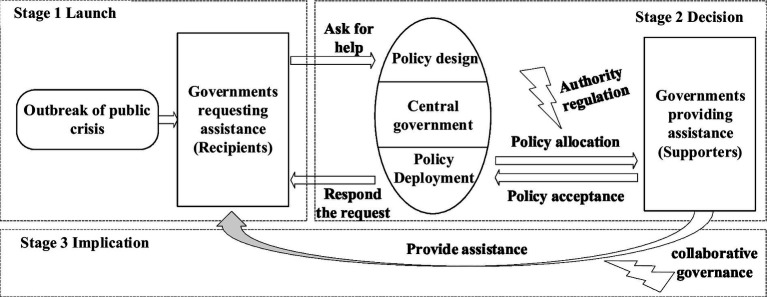
Operational model of collaborative governance under the authoritative regulation.

As shown in Figure 1, we have refined the original collaborative governance model to elaborate a novel framework for collaborative governance under the authoritative regulation. The initial stage, called the launch stage, is characterized by the outbreak of a public crisis, triggered by natural disasters or public health incidents, which initially manifests in one or more districts. Local governments are often ill-prepared for such emergencies due to the abrupt nature of the outbreak, leading to the spread of the crisis. As the impact of the major public crisis intensifies, the capacity of local governments to manage the situation diminishes, increasing the risk of adverse spillovers. At the same time, secondary crises, such as economic downturns (60), begin to emerge, compounding the crisis. As public crises exceed the capacity of local governments, and as the crisis expands, higher levels of government become involved. Reflecting the situation to higher levels of government and requesting assistance becomes the only viable option for local governments.

Moving on to the second stage, the decision step involves the assessment by the higher-level government of whether to provide assistance to the requesting jurisdiction. This decision should be based on a comprehensive assessment of the request, based on the specific circumstances of the crisis, to determine the appropriateness of activating pairing assistance. Following the evaluation, the higher-level government should set explicit objectives for cooperative governance in response to the major public crisis facing the requesting government and proceed to enact an appropriate cooperative governance. The central government, exercising its authority and taking into account the specific circumstances of each local government, designates supporters for crisis governance. Each supporting government is assigned specific tasks, establishing a one-to-one cooperative relationship between the supporting and requesting parties. At this point, collaborative governance moves into the implementation phase, as the two basic requirements—well-defined objectives and well-defined relationships—have been established.

Throughout the implementation stage of collaborative governance, the supporting government provides the recipient with governance resources, including personnel, material and financial support. It also sets up a coordination mechanism to facilitate collaboration around specific tasks. Throughout this process, the central government monitors the interactions between local governments and mediates any conflicts that may arise, thereby ensuring the integrity and stability of the cooperative relationship. The existing cooperative relationship is continuously refined in response to the evolving situation to ensure the seamless fulfillment of crisis governance objectives. It is important to note that collaborative governance in public crises is often of a temporary nature. Nevertheless, the collaborative efforts during this period can lay the foundation for a lasting cooperative relationship between the parties, which can have a positive impact on fostering ongoing interaction between them.

## 5 Conclusion and discussion

### 5.1 Key results

Pairing assistance in public crises is a quintessential form of collaborative governance under authoritative regulation. In contemporary society, major public crises span multiple academic disciplines and transcend various boundaries (61). For example, COVID-19 has rigorously tested the emergency preparedness efforts of governments at all levels, especially at the local level (62). This study, seeks to synthesize previous research on pairing support during public crises, using collaborative governance under authoritative regulation as a conceptual framework. The validity and applicability of this framework is substantiated through case reviews and discussions, and offers utility and guidance for future research in the area of central-local partnerships and crisis response.

This research extends the understanding of the pairing assistance system by examining the practical outcomes of Jiangsu Province’s assistance to Huangshi City in Hubei Province through a detailed case study. It also introduces an analytical framework relevant to collaborative governance under authoritative regulation. The argument argues in this paper is that crisis response requires the guidance of a central authority in conjunction with the cooperation of local governments. This process is motivated by three key factors: the vertical intervention of the crisis (including the intensification of political discourse and the reinforcement of principal-agent relationships), the inevitability of horizontal cooperation (including the nature of transboundary crises, the foundation for cooperation, and the accumulation of practical experience), and the policy allocation and incentives within the bureaucracy (including policy allocation mechanisms and the interplay of incentives and restraints).

The three stages of cooperative governance, based on authoritative regulation, operate in a sequential manner to enhance the effectiveness of crisis response. Given the limited and uneven distribution of crisis management resources across regions, local governments may find it difficult to manage major public crises independently, necessitating collaboration with other government agencies. Although local governments may have a common interest in crisis response and a willingness to cooperate, the collaborative management of public crises may be hampered by factors such as a lack of willingness to cooperate, local opportunism and the risks associated with cooperation, which make it difficult to reach agreements. The authoritative government, typically the central government, can facilitate the resolution of barriers to collaborative governance among local governments. By setting governance objectives and delineating specific governance tasks through its authority, the central government can transform the potential cooperative tendency of local governments into actionable behavior, thereby effectively achieving the cross-regional deployment of crisis governance resources.

In conducting this case study, we found that a distinctive feature of pairing assistance during the epidemic is the delegation of resource allocation authority from the central to the local level, with specific assistance plans communicated and negotiated between the providing and receiving jurisdictions. In some provinces, such as Jiangsu, cities at the prefecture level are responsible for implementing assistance after receiving requests for assistance. Hubei Province, the center of the epidemic, is located in the central region of China, and Wuhan is a strategic crossroads linking several provinces. The high volume of population movement facilitates the rapid spread of COVID-19, particularly as it coincides with the Chinese Spring Festival, a period characterized by mass migration in a short period of time. The uncontrollable nature of COVID-19 is thus magnified exponentially. Given the severity of the epidemic and its propensity for widespread transmission, the task of epidemic prevention transcends provincial boundaries and requires assistance from other provinces. Subjectively, the implementation of pairing assistance in the wake of the epidemic outbreak has a strong political significance in China. Faced with the sudden emergence of COVID-19, the Party Central Committee and the State Council, adhering to the principle of prioritizing people’s well-being and lives, are compelled to act swiftly to contain the spread of the virus, alleviate social panic and minimize its negative impact on the functioning of society. Systematically, the central government is exercising its authority to mobilize local governments to form pairing assistance, thereby achieving an efficient and rational allocation of resources across different regions and effectively responding to major public crises such as COVID-19.

### 5.2 Policy side effects

When dealing with major public crises and emergencies, China’s model of pairing assistance is undoubtedly a viable tool for other nations to consider. From this case study and historical precedents, it is clear that targeted assistance can significantly aid the response to public crises. However, it is important to recognize that no system is universally effective. The system of pairing assistance following a public crisis is no exception, and it may have certain side effects.

First, there is uncertainty about the effectiveness of pairing assistance. While previous research has largely emphasized its positive outcomes, a practical perspective reveals that pairing assistance can facilitate the rapid recovery of production and daily life in recipient areas in the short term. However, it is imperative to acknowledge and address potential problems. For example, research suggests that the intensity of aid provided by coastal provinces following the Wenchuan earthquake exceeded the standards set by the central government, potentially leading to the “Dutch disease” in the short term due to local competition (63). The influx of large-scale aid projects has resulted in a surge in prices in the post-disaster reconstruction areas.

Second, the application of pairing assistance in epidemic response presents specific challenges that cannot be overlooked. For example, when the aid-providing jurisdiction itself is affected by an epidemic, the outflow of epidemic prevention forces and resources may compromise the local ability to manage the crisis. The process of cross-regional mobility may give rise to new outbreaks of the epidemic. Particularly at the outset of COVID-19, the highly contagious nature of the virus poses a risk of infection during the supply of aid, the deployment of aid workers to affected sites, and the transfer of close and sub-close contact cases within aid locations. In additioon, the assistance agencies assume considerable risks in the execution of their duties, which extends beyond the provision of financial and material support to necessitate courage and perseverance.

Third, there is an absence of comprehensive legal protection for pairing assistance. The current practice of pairing assistance resembles a political task promoted by the Party Central Committee and the State Council to be undertaken by local governments, rather than a legally binding obligation. There is currently no clear legal document in China to confirm and limit this policy.

Fourth, the sustainability of pairing assistance remains a subject of debate. From a practical point of view, pairing assistance depends on pressure mechanisms such as political authority, target setting and accountability to encourage local governments to fulfill their responsibilities. Local governments are expected to comply with administrative directives from superior and central government authorities. The provision of support, including medical supplies, daily necessities, and medical personnel, often involves substantial unpaid investments with limited prospects for return, leading to potential sustainability challenges. As a result, the initiative and enthusiasm of the supporting parties may be constrained, posing challenges to the long-term viability of the pairing assistance model.

### 5.3 Limitation and future research

Due to the constraints of information availability, material resources, and the scope of this study, certain aspects were not discussed in detail. China has demonstrated considerable success in managing epidemic prevention efforts through the mechanism of pairing assistance. However, this model has distinctive Chinese characteristics, and its replicability in other countries around the world may be influenced by a variety of factors, including cultural context and political infrastructure. Despite these considerations, the concept of pairing assistance offers a viable and optional strategy for the effective prevention and control of similar events in the future. The unique characteristics of this approach, while shaped by the specific context of China, may still provide valuable insights and a potential framework for international cooperation in the face of public health crises. Further research is encouraged to explore the adaptability and applicability of pairing assistance in diverse settings, thereby contributing to the global repository of knowledge on crisis management and cooperative governance.

## Data Availability

The original contributions presented in the study are included in the article/supplementary material, further inquiries can be directed to the corresponding author.

## References

[1] Huang R Yao X Chen Z Li W Yan H. The impact of China’s paired assistance policy on the COVID-19 crisis—An empirical case study of Hubei Province. Front Public Health. (2022) 10:885852. doi: 10.3389/fpubh.2022.885852, PMID: 35712299 PMC9196880

[2] Lyu S Qian C McIntyre A Lee C-H. One pandemic, two solutions: comparing the U.S.-China response and health priorities to COVID-19 from the perspective of “two types of control”. Healthcare. (2023) 11:1848. doi: 10.3390/healthcare11131848, PMID: 37444682 PMC10341116

[3] Feng Y Li Q Tong X Wang R Zhai S Gao C. Spatiotemporal spread pattern of the COVID-19 cases in China. PLoS One. (2020) 15:e0244351. doi: 10.1371/journal.pone.0244351, PMID: 33382758 PMC7775067

[4] Xinhua Daily Newspaper. Jiangsu Province sends first batch of 310-member medical support team to Huangshi city in Hubei province (in Chinese). (2020). Available at: http://lsj.jiangsu.gov.cn/art/2020/2/12/art_74732_8968960.html (Accessed March 1, 2024).

[5] Modern Express. The people of Huangshi escorted the Jiangsu medical support team back home (in Chinese). (2020). Available at: https://news.ifeng.com/c/7vD7ciVl6bF (Accessed March 1, 2024).

[6] China News. Jiangsu medical volunteer team returns safely to Jiangsu province [in Chinese]. (2020). Available at: https://www.chinanews.com/tp/2020/03-28/9140639.shtml (Accessed October 10, 2024).

[7] Kettl DF. States divided: the implications of American federalism for COVID-19. Public Adm Rev. (2020) 80:595–602. doi: 10.1111/puar.13243, PMID: 32836439 PMC7280573

[8] Mallinson DJ. Cooperation and conflict in state and local innovation during COVID-19. Am Rev Public Adm. (2020) 50:543–50. doi: 10.1177/0275074020941699

[9] Joyce PG Suryo PA. Government responses to the coronavirus in the United States: immediate remedial actions, rising debt levels and budgetary hangovers. JPBAFM. (2020) 32:745–58. doi: 10.1108/JPBAFM-07-2020-0111

[10] Mao Y. Political institutions, state capacity, and crisis management: a comparison of China and South Korea. Int Polit Sci Rev. (2021) 42:316–32. doi: 10.1177/0192512121994026

[11] Kim Y Poncelet J-L Min G Lee J Yang Y. COVID-19: systemic risk and response management in the Republic of Korea. Prog Disaster Sci. (2021) 12:100200. doi: 10.1016/j.pdisas.2021.100200, PMID: 34493999 PMC8413094

[12] Yoo KJ Kwon S Choi Y Bishai DM. Systematic assessment of South Korea’s capabilities to control COVID-19. Health Policy. (2021) 125:568–76. doi: 10.1016/j.healthpol.2021.02.011, PMID: 33692005 PMC7927652

[13] Chen T Wang Y Hua L. “Pairing assistance”: the effective way to solve the breakdown of health services system caused by COVID-19 pandemic. Int J Equity Health. (2020) 19:68. doi: 10.1186/s12939-020-01190-8, PMID: 32414384 PMC7226711

[14] Pan X Ojcius DM Gao T Li Z Pan C Pan C. Lessons learned from the 2019-nCoV epidemic on prevention of future infectious diseases. Microbes Infect. (2020) 22:86–91. doi: 10.1016/j.micinf.2020.02.004, PMID: 32088333 PMC7102576

[15] Smith W Dowell J. A case study of co-ordinative decision-making in disaster management. Ergonomics. (2000) 43:1153–66. doi: 10.1080/00140130050084923, PMID: 10975178

[16] Raju E Van Niekerk D. Intra-governmental coordination for sustainable disaster recovery: a case-study of the Eden District municipality, South Africa. Int J Disaster Risk Reduct. (2013) 4:92–9. doi: 10.1016/j.ijdrr.2013.03.001

[17] Steigenberger N. Organizing for the big one: a review of case studies and a research agenda for multi-agency disaster response. J Contingencies Crisis Manag. (2016) 24:60–72. doi: 10.1111/1468-5973.12106

[18] Kapucu N Hu Q Harmon M Toro P. Coordinating non-established disaster relief groups: a case study of hurricane Irma in Florida. United States *Disasters*. (2021) 45:717–37. doi: 10.1111/disa.12439, PMID: 32342534

[19] Chao CNG Emily CWS Forlin C Ho FC. Improving teachers’ self-efficacy in applying teaching and learning strategies and classroom management to students with special education needs in Hong Kong. Teach Teach Educ. (2017) 66:360–9. doi: 10.1016/j.tate.2017.05.004

[20] Hasan MB Mahi M Sarker T Amin MR. Spillovers of the COVID-19 pandemic: impact on global economic activity, the stock market, and the energy sector. JRFM. (2021) 14:200. doi: 10.3390/jrfm14050200

[21] Gao J Zhang P. Mechanisms of the Chinese Government’s efforts to fight COVID-19: integration of top-down interventions and local governance. Health Secur. (2022) 20:348–56. doi: 10.1089/hs.2021.0161, PMID: 35787156

[22] Zhong K Liu Y Christensen T. Crisis coordination in centralized regimes: explaining China’s strategy for combatting the COVID-19 pandemic. Int Public Manag J. (2022) 25:1131–50. doi: 10.1080/10967494.2022.2073411

[23] Zhao Y Cheng S Yu X Xu H. Chinese Public’s attention to the COVID-19 epidemic on social media: observational descriptive study. J Med Internet Res. (2020) 22:e18825. doi: 10.2196/18825, PMID: 32314976 PMC7199804

[24] Tisdell CA. Economic, social and political issues raised by the COVID-19 pandemic. Econ Anal Policy. (2020) 68:17–28. doi: 10.1016/j.eap.2020.08.002, PMID: 32843816 PMC7440080

[25] Qian J Liu Z Du Y Wang N Yi J Sun Y. Multi-level inter-regional migrant population estimation using multi-source spatiotemporal big data: a case study of migrants in Hubei Province during the outbreak of COVID-19 in Wuhan In: Shaw S-L Sui D, editors. *Mapping COVID-19 in space and time*. Human dynamics in smart cities. Cham: Springer International Publishing (2021). 169–88.

[26] Zhu Z Liu Q Jiang X Manandhar U Luo Z Zheng X. The psychological status of people affected by the COVID-19 outbreak in China. J Psychiatr Res. (2020) 129:1–7. doi: 10.1016/j.jpsychires.2020.05.026, PMID: 32526513 PMC7255091

[27] Pei J De Vries G Zhang M. International trade and Covid-19: City-level evidence from China’s lockdown policy. J Reg Sci. (2022) 62:670–95. doi: 10.1111/jors.12559, PMID: 34548696 PMC8447424

[28] Xiong Y Wang Y Chen F Zhu M. Spatial statistics and influencing factors of the COVID-19 epidemic at both prefecture and county levels in Hubei Province, China. IJERPH. (2020) 17:3903. doi: 10.3390/ijerph17113903, PMID: 32486403 PMC7312640

[29] You M Wu Z Yang Y Liu J Liu D. Spread of coronavirus 2019 from Wuhan to rural villages in the Hubei Province. Open Forum Infect Dis. (2020) 7:ofaa228. doi: 10.1093/ofid/ofaa228, PMID: 33117853 PMC7313863

[30] Zhang J Hayashi Y. Research frontier of COVID-19 and passenger transport: a focus on policymaking. Transp Policy. (2022) 119:78–88. doi: 10.1016/j.tranpol.2022.02.014, PMID: 35233151 PMC8874153

[31] Yang Z Zeng Z Wang K Wong S-S Liang W Zanin M. Modified SEIR and AI prediction of the epidemics trend of COVID-19 in China under public health interventions. J Thorac Dis. (2020) 12:165–74. doi: 10.21037/jtd.2020.02.64, PMID: 32274081 PMC7139011

[32] Ye Q Zhou J Wu H. Using information technology to manage the COVID-19 pandemic: development of a technical framework based on practical experience in China. JMIR Med Inform. (2020) 8:e19515. doi: 10.2196/19515, PMID: 32479411 PMC7282474

[33] Chen C Liu R. How public confidence was established during the COVID-19 pandemic by Chinese media: a corpus-based discursive news value analysis. Front Public Health. (2022) 10:1012374. doi: 10.3389/fpubh.2022.1012374, PMID: 36388262 PMC9640770

[34] Zhu S Feng S Ning X Zhou Y. Analysis of China’s fight against COVID-19 from the perspective of policy tools—policy capacity. Front Public Health. (2022) 10:951941. doi: 10.3389/fpubh.2022.951941, PMID: 36203691 PMC9531593

[35] People’s Daily Online (PRC Newspaper). The last medical team from Jiangsu to Hubei returned to Jiangsu [in Chinese]. (2020). Available at: http://js.people.com.cn/n2/2020/0413/c360303-33945501.html (Accessed October 12, 2024).

[36] Mertha AC. China’s “soft” centralization: shifting Tiao/Kuai authority relations. China Q. (2005) 184:791–810. doi: 10.1017/S0305741005000500

[37] Liu J Guo Y An S Lian C. A study on the mechanism and strategy of cross-regional emergency cooperation for natural disasters in China—based on the perspective of evolutionary game theory. IJERPH. (2021) 18:11624. doi: 10.3390/ijerph182111624, PMID: 34770138 PMC8583056

[38] Hung S-C Chang S-C. Framing the virus: the political, economic, biomedical and social understandings of the COVID-19 in Taiwan. Technol Forecast Soc Chang. (2023) 188:122276. doi: 10.1016/j.techfore.2022.122276, PMID: 36594080 PMC9797412

[39] Xu Y Ge W Liu G Su X Zhu J Yang C. The impact of local government competition and green technology innovation on economic low-carbon transition: new insights from China. Environ Sci Pollut Res. (2022) 30:23714–35. doi: 10.1007/s11356-022-23857-1, PMID: 36327068 PMC9630813

[40] Xian M Zhao C Zhou Y. From bureaucratic coordination to a data-driven model: transformation and capacity building of community-based prevention and control of public health events. IJERPH. (2022) 19:8238. doi: 10.3390/ijerph19148238, PMID: 35886091 PMC9323052

[41] Ansell C Gash A. Collaborative governance in theory and practice. J Public Adm Res Theory. (2008) 18:543–71. doi: 10.1093/jopart/mum032

[42] Emerson K Nabatchi T Balogh S. An integrative framework for collaborative governance. J Public Adm Res Theory. (2012) 22:1–29. doi: 10.1093/jopart/mur011

[43] Abd Samat AH Abdul Rashid A Mohd Yunus NA Salim AMH Musa H. A Malaysian medical non-governmental Organization’s (NGO) experience in the emergency response for COVID-19, using the whole-of-society collaborative concept. Disaster Med Public Health Prep. (2022) 16:2665–8. doi: 10.1017/dmp.2021.106, PMID: 33820586 PMC8220015

[44] Chaudhuri K Chakrabarti A Lima JM Chandan JS Bandyopadhyay S. The interaction of ethnicity and deprivation on COVID-19 mortality risk: a retrospective ecological study. Sci Rep. (2021) 11:11555. doi: 10.1038/s41598-021-91076-8, PMID: 34078992 PMC8172854

[45] Mondal S Van Belle S Bhojani U Law S Maioni A. Policy processes in multisectoral tobacco control in India: the role of institutional architecture, political engagement and legal interventions. Int J Health Policy Manag. (2021):1. doi: 10.34172/ijhpm.2021.66, PMID: 34380195 PMC9808220

[46] Li X Jiang H Liang X. Early stage risk identification and governance of major emerging infectious diseases: a double-case study based on the Chinese context. RMHP. (2023) 16:635–53. doi: 10.2147/RMHP.S400546, PMID: 37056713 PMC10089271

[47] Huang L Zhang J Huang Q Cui R Chen J. In-hospital major adverse cardiovascular events after primary percutaneous coronary intervention in patients with acute ST-segment elevation myocardial infarction: a retrospective study under the China chest pain center (standard center) treatment system. BMC Cardiovasc Disord. (2023) 23:198. doi: 10.1186/s12872-023-03214-x, PMID: 37069503 PMC10111847

[48] Tang J Li S. How do environmental regulation and environmental decentralization affect regional green innovation? Empirical research from China. IJERPH. (2022) 19:7074. doi: 10.3390/ijerph19127074, PMID: 35742320 PMC9222837

[49] Tang G Lin M Xu Y Li J Chen L. Impact of rating and praise campaigns on local government environmental governance efficiency: evidence from the campaign of establishment of national sanitary cities in China. PLoS One. (2021) 16:e0253703. doi: 10.1371/journal.pone.0253703, PMID: 34166450 PMC8224862

[50] Aljukhadar M. National Vulnerability to pandemics: the role of macroenvironmental factors in COVID-19 evolution. J Environ Public Health. (2022) 2022:1–10. doi: 10.1155/2022/9524407, PMID: 35342433 PMC8944916

[51] Carling A. Market, state, and community: theoretical foundations of market socialism. By David miller. Oxford: Clarendon, 1989. 359p. $59.00. Am Polit Sci Rev. (1990) 84:1359–60. doi: 10.2307/1963284

[52] Li X Yang X Gong L. Evaluating the influencing factors of urbanization in the Xinjiang Uygur autonomous region over the past 27 years based on VIIRS-DNB and DMSP/OLS nightlight imageries. PLoS One. (2020) 15:e0235903. doi: 10.1371/journal.pone.0235903, PMID: 32697778 PMC7375535

[53] Zhu G Li Y Sun Z Kanae S. Response of vegetation to submergence along Jingjiang reach of the Yangtze River. PLoS One. (2021) 16:e0251015. doi: 10.1371/journal.pone.0251015, PMID: 33961662 PMC8104387

[54] Abbas HSM Xu X Sun C Abbas S. Impact of administrative state capacity determinants on sustainable healthcare. Heliyon. (2023) 9:e18273. doi: 10.1016/j.heliyon.2023.e18273, PMID: 37539303 PMC10395479

[55] Li Y Fang X. Officials’ promotion expectation, corporate strategic deviance and corporate growth in China: the moderating effect of corporate ownership. PLoS One. (2023) 18:e0284872. doi: 10.1371/journal.pone.0284872, PMID: 37624770 PMC10456177

[56] Ma Q Zhang Y Samual A Hu F Touns M. Does the creation of healthy cities promote municipal solid waste management? Empirical research in 284 cities in China. Front Public Health. (2022) 10:1030283. doi: 10.3389/fpubh.2022.1030283, PMID: 36388356 PMC9659738

[57] Badman RP Wang AX Skrodzki M Cho H-C Aguilar-Lleyda D Shiono N. Trust in institutions, not in political leaders, determines compliance in COVID-19 prevention measures within societies across the globe. Behav Sci. (2022) 12:170. doi: 10.3390/bs12060170, PMID: 35735380 PMC9219766

[58] Bryson JM Crosby BC Bloomberg L. Public value governance: moving beyond traditional public administration and the new public management. Public Adm Rev. (2014) 74:445–56. doi: 10.1111/puar.12238

[59] Bennett S Glandon D Rasanathan K. Governing multisectoral action for health in low-income and middle-income countries: unpacking the problem and rising to the challenge. BMJ Glob Health. (2018) 3:e000880. doi: 10.1136/bmjgh-2018-000880, PMID: 30364411 PMC6195144

[60] Shalitin S Phillip M Yackobovitch-Gavan M. Changes in body mass index in children and adolescents in Israel during the COVID-19 pandemic. Int J Obes. (2022) 46:1160–7. doi: 10.1038/s41366-022-01092-5, PMID: 35173280 PMC8852981

[61] Smith D Fischbacher M. The changing nature of risk and risk management: the challenge of borders, uncertainty and resilience. Risk Manag. (2009) 11:1–12. doi: 10.1057/rm.2009.1

[62] Dzigbede KD Gehl SB Willoughby K. Disaster Resiliency of U.S. Local Governments: Insights to strengthen local response and recovery from the COVID-19 pandemic. Public Adm Rev. (2020) 80:634–43. doi: 10.1111/puar.13249, PMID: 32836460 PMC7300832

[63] Bulte E Xu L Zhang X. Post-disaster aid and development of the manufacturing sector: lessons from a natural experiment in China. Eur Econ Rev. (2018) 101:441–58. doi: 10.1016/j.euroecorev.2017.10.019

